# Emerging roles of CREST superfamily members in lipid metabolism

**DOI:** 10.1016/j.jlr.2026.101117

**Published:** 2026-08-04

**Authors:** Takuma Takagaki, Tomoyoshi Shingaki, Nozomu Kono, Junken Aoki

**Affiliations:** Laboratory of Health Chemistry, Graduate School of Pharmaceutical Sciences, the University of Tokyo, Tokyo, Japan

**Keywords:** CREST, PAQR, adiponectin receptor, phospholipase, ceramidase, phospholipid, ceramide, GPI

## Abstract

CREST superfamily members are a series of multi-transmembrane proteins conserved across a wide range of species. There are approximately 20 members in mammals. CREST superfamily members have been assigned various functions, including those of receptors, transporters, enzymes, and membrane fusion factors. Recent functional and structural analyses of CREST superfamily members have revealed that all members possess a structure resembling an enzymatic active site that catalyzes hydrolysis reactions. Furthermore, it is becoming increasingly clear that they interact with various membrane lipids and are involved in lipid metabolism. This review summarizes current knowledge of the structure and function of each CREST superfamily member and explores their potential as lipid-metabolizing enzymes.

The CREST superfamily was first proposed in 2011 by the research group of Grishin based on sequence similarity, and its members are a series of multitransmembrane-spanning proteins with putative catalytic activity within the cell membrane (1). The five letters of ‘CREST’ are derived from the letters of ‘alkaline Ceramidase’, ‘PAQR Receptor’, ‘per1’, ‘Sid-1’, and ‘Tmem8’. Figure 1 shows the conservation of CREST family members across various species. Homologs of the CREST family members are found in a wide range of species, from yeast to mammals (Fig. 1A). In humans, there are 20 members, including 11 members of the progestin and adipoQ receptor (PAQR) family (Fig. 1B, C). A phylogenetic tree is presented to provide an overview of the relationships among the 20 CREST superfamily members in humans (Fig. 1B). In mice, 20 members are highly conserved. In zebrafish, while some members exist in two forms, 19 members are conserved, with the exception of Sid1 (Fig. 1A, B). Thus, CREST family members are highly conserved in higher organisms, including fish and mammals. On the other hand, in lower organisms, while some members are conserved across species, others are not. For example, homologs of PAQR1/AdipoR1 and PAQR2/AdipoR2 are present in all species (Fig. 1A, B). However, in *Drosophila*, *C. elegans*, and yeast, only one of these homologs appears to be present. PAQR3 and 4 appear to be conservatively conserved across a wide range of species, whereas PAQR5–9—with the exception of PAQR8—are conserved only in higher vertebrates. Homologs of PAQR10 and 11 are also present in *Drosophila* and *C. elegans* but are absent in yeast. Among CREST family members outside the PAQR family, the ACER subfamily is relatively well conserved across species, whereas the SID, PGAP, and TMEM8 subfamilies show low levels of conservation across species.

**Fig. 1:**
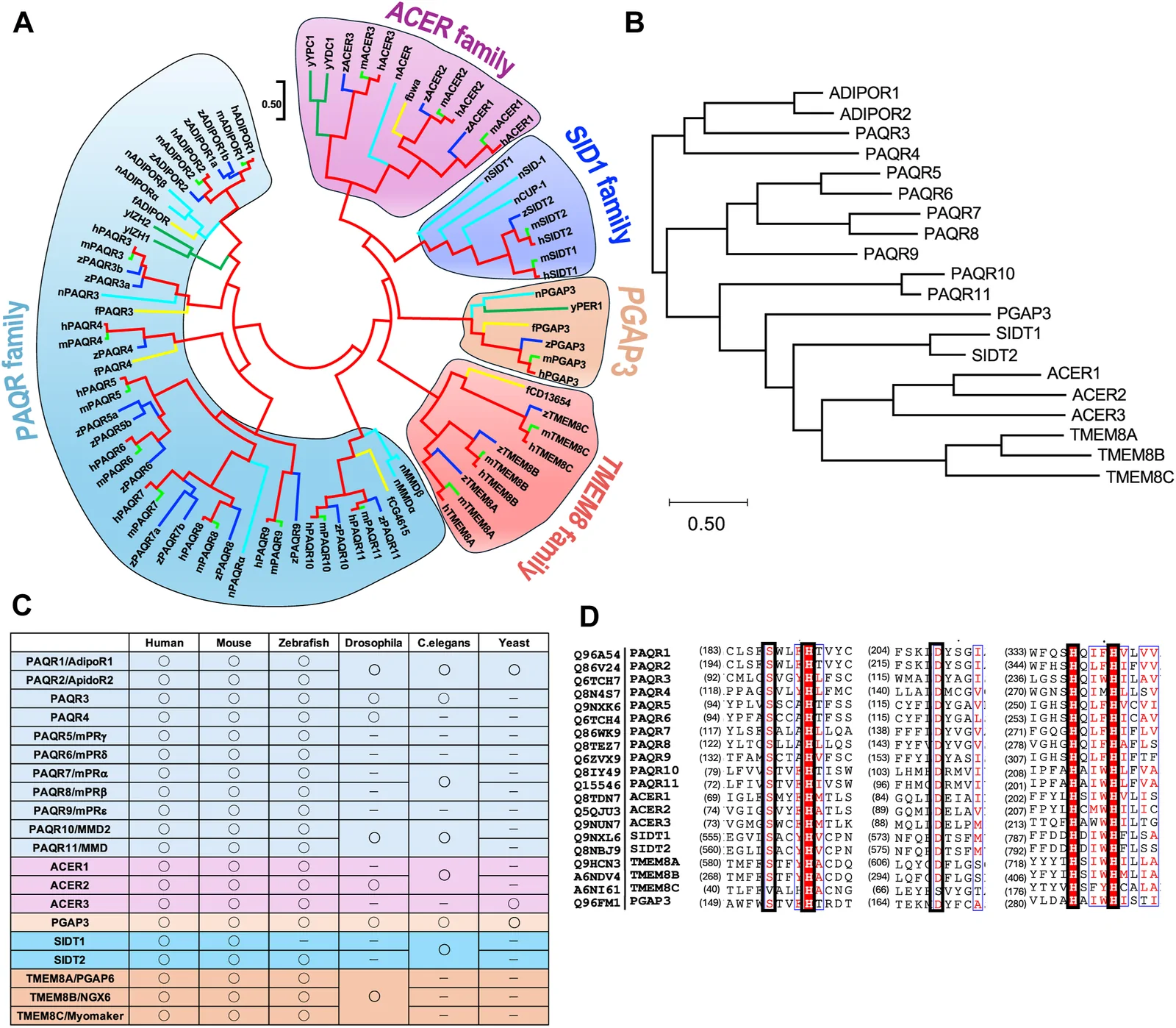
Phylogenetic tree and multiple sequence alignment of CREST superfamily members. A: Detailed phylogenetic analysis of CREST superfamily members across various species. The small letters “h”, “m”, “z”, “f”, “n” and “y” at the beginning of protein names indicate the origins of amino acid sequences, *i.e.*, human (*H. sapiens*), mouse (*M. musculus*), zebrafish (*D. rerio*), fruit fly (*D. melanogaster*), nematode (*C. elegans*) and yeast (*S. cerevisiae*), respectively. Note that in zebrafish, due to gene duplication, some genes exist in duplicate. The PAQR, ACER, SID, GPAP3 and TMEM8 branches are shown in light blue, purple, blue, orange and red, respectively. Scale bar indicates 0.5 amino acid substitutions per site. B: Phylogenetic tree of 20 human CREST superfamily members. C: The presence of CREST superfamily members across various species. “○” indicates that the member is present in the species. In lower organisms, it is sometimes not clear which member in higher organisms corresponds to a given member. In such cases, “○” is marked across multiple members. D: Multiple sequence alignment of 20 CREST superfamily members. The sequences are shown to highlight the three His residues, one Ser residue, and one Asp residue (H3-S-D) that constitute the active site. Note that not all amino acids are shown. Starting residue numbers are shown in brackets. The amino acids comprising H3–S–D are enclosed in black boxes. These figures were generated using protein sequences obtained from UniProt and National Center for Biotechnology Information. Sequence alignment was performed using MAFFT v7, the phylogenetic tree was constructed using MEGA 12, and the alignment was visualized using ESPript 3.2.

To date, various biochemical and biological functions have been assigned to each member, including ligand receptors [PAQR1/AdipoR1, PAQR2/AdipoR2, PAQR5 (membrane progesterone receptor γ, mPRγ), PAQR6 (mPRδ), PAQR7 (mPRα), PAQR8 (mPRβ), and PAQR9 (mPRϵ)], a cell differentiation marker (PAQR11/MMD), a myocyte fusion promoter (TMEM8C/Myomaker), a tumor suppressor (TMEM8B/NGX6), lipolytic enzymes (PAQR1/AdipoR1, PAQR2/AdipoR2, alkaline ceramidase, PER-1, and SIDT1), phospholipid metabolism (PAQR1/AdipoR1, PAQR2/AdipoR2, PAQR9, PAQR11/MMD, SIDT1, and SIDT2), and intracellular transport of dsRNA (SIDT1) (Table 1). The most distinctive feature of the CREST superfamily is that all members possess a motif (H3-S-D) between transmembrane segments, which is presumed to constitute the catalytic active site (Fig. 1D). In this review, we summarize the structure of the CREST superfamily members, with a particular focus on their catalytically active domains, and discuss the biological functions of each member, including their roles as lipid-metabolizing enzymes. Since the first publication of a review on the CREST family in 2011 (1), significant progress has been made regarding the biological functions and, in particular, the tertiary structures of each member. This review summarizes these findings while also incorporating findings from before 2011.

**Table 1:** CREST family members: Overview

Name	Gene Name	Length (aa)	Expression Pattern	Subcellular Localization	Knock out Animals Phenotype	Biological Function	Change in lipid Profile in Mutant	Remarks	Reference
PAQR1/AdipoR1	ADIPOR1, PAQR1,TESBP1A, CGI-45	375	ubiquitous; predominant in eye, brain, and whole blood	PM, ER (transient overexpression cells)	retinal degeneration impaired kidney function exacerbation of insulin resistanceImpaired thermogenesis	activation of the AMPK pathway ceramidase	DHA-phospholipids↓	embryonic lethality in Adipor1/2 DKO mice	(2, 3, 4, 5, 6, 7, 8)
PAQR2/ApidoR2	ADIPOR2, PAQR2	386	ubiquitous; predomnant in adipose tissue	PM, ER (transient cells)	male infertility impaired thermogenes	activation of PPARα pathway ceramidase	Palmitate-phospholipids↑	embryonic lethality in Adipor1/2 DKO mice	(2, 7, 9)
PAQR3	PAQR3, RKTG	311	ubiquitous	Golgi	enhances VEGF-mediated angiogenesis promotion of skin wound healing in diabetic mice	tumor suppression regulation of cholesterol homeostasis by anchoring Scap/SREBPnegative regulation of the PI3K/AKT signaling pathway	hepatic cholesterol↓		(10, 11, 12, 13, 14, 15, 16)
PAQR4	PAQR4	273	ubiquitous; except in the breast	Golgi	metabolic capacity of adipocytes is improved in obesity.	ceramidase? mediation of the stability of ceramide synthetases	sphingolipids↓		(2, 10, 17, 18)
PAQR5/mPRg	PAQR5, MPRG	330	kidney (nephron), colon	PM, ER	shrunked olfactory rosette impaired oocyte maturation	inhibition of the JAK/STAT3 signaling pathway	n.d.		(19, 20, 21, 22, 23, 24, 25, 29)
PAQR6/mPRd	PAQR6, PRdelta	344	brain	PM	n.d.	involved in angiogenesis and pluripotent stem cell differentiation	n.d.		(10, 22, 26, 27)
PAQR7/mPRa	PAQR7, MPRA, PGLP, mSR	346	kidney (proximal tubules), placenta, testis, ovary	PM, ER	impaired oocyte maturation prolonged estrous cycle, reduced follicular growth, increased the number of atresia follicles, and decreased the concentrations of E2 and AMH	activation of ERK1/2 signaling	n.d.		
PAQR8/mPRb	PAQR8, C6orf33, LMPB1, MPRB	354	brain (pituitary), spinal cord, testis, ovary	PM, ER	impaired oocyte maturation	activation of maturation promoting factor (e.g. Cdk1,cyclin B) induction of meiosis through coupling with ABHD2 regulation of the decidualization process	generation of lipid mediators(e.g. S1P) via coupling with ABHD2		(19, 21, 24, 26, 31, 32, 27, 28, 29, 30, 33, 34, 35)
PAQR9/mPRe	PAQR9	377	brain, oocyte	PM, ER	reduced chorion elevation a high percentage of abnormal embryos improves hyperglycemia and glucose tolerance in diabetic mice	meditation of insulin secretion in β cells in vitro under stress conditions	Arachidonic acid metabolities↓PUFA-containing PL↓		(22, 36, 37, 29, 38)
PAQR10/MMD2	MMD2,PAQR10	270	brain, testis	Golgi	no abnormalitieies in testis development	mediation of Ras signaling in the Golgi apparatus downstream regulation of sex-determining genes	n.d.	aggressive periodontitis in a specific mutation	(10, 39, 40, 41)
PAQR11/MMD	MMD,PAQR11	238	ubiquitous	Golgi	imparied macrophage differentiation increased apoptosis in didfferentiated BMDMs, amelioration of rheumatoid arthritis pathology resistant to high-fat diet-induced obesity, enhancement of lipolysis	mediation of Ras signaling in the Golgi apparatus macrophage differentiation interaction with LPLAT11 and ACSL4 to promote the synthesis of Arachidonic acid-containing PI	Arachidonic acid-containing PI↓		(10, 39, 42, 43, 44, 45)
ACER1	ACER1, ASAH3	264	skin	ER	progressive Alopecia	regulation of epidermal and appendage homeostasis	ceramide↑ sphingoid base↑		(46, 47, 48)
ACER2	ACER2, ASAH3L, PP11646	275	ubiquitous, placenta	Golgi	increased susceptibility for lithium-induced nephrogenic diabetes insipidus	induction of autophagy and apoptosis in cancer cells production of sphingosine-1-phosphate (S1P)	SHP↓ S1P↓ dhSph↓ dhS1P↓ in blood	N-terminus requirement for Golgi localization, ceramidase activity, and protein glycosylationp53-dependent transcriptional regulationACER2 mRNA stabilization by YTH N6-methyladenosine RNA binding protein 2	(47, 49, 50, 51, 52, 53, 54)
ACER3	ACER3, APHC, PHCA	267	ubiquitous	ER, Golgi	purkinje cell degeneration in the cerebellum deficits in motor coordination and balance	regulation of ceramides and sphingosine-1-phosphate homeostasis	ceramides,C18:1-monohexosylceramid↑S1P↓ in brain sphingolipids↑	reduced ACER3 activity due to a homozygous E33G mutation in leukodystrophy patients	(46, 55, 56, 57, 58, 59)
PGAP3/Per-1	PGAP3, CAB2, PERLD1, UNQ546/PRO1100	320	ubiquitous	Golgi	reduced birth rate of males growth retardation abnormal reflexes excessive activation of T cells	deacylation of unsaturated fatty acids at the sn-2 position of GPI	unsaturated GPI↑		(60, 61)
SIDT1	SIDT1	827	stomach	PM, ER, Endolysosomal membrene	diminished dietary miRNA absorption reduced type I IFN production following HSV infection	cellular uptake of siRNA and miRNAs cellular uptake of cholesterol conjugated siRNAs in liver HepG2 cells uptake of exogenous microRNAs by gastric pit cells cholesterol transport	n.d.		(62, 63, 64, 65)
SIDT2	SIDT2, CGI-40, PSEC0072, UNQ685/PRO1325	832	ubiquitous	ER, Lysosome and endolysosomal membrane	accumulation of lipid droplets in liver reduced type I IFN production following HSV infection	cholesterol transport	serum TG↑ serum FFA↑ lipid droplets in liver ↑		(62, 66, 67, 68, 69)
TMEM8A/PGAP6	PGAP6, TMEM6, TMEM8, TMEM8A	771	ubiquitous	PM	anterior–posterior axis defect	Nodal signaling through CRIPTO shedding	n.d.		(70, 71)
TMEM8B/NGX6	TMEM8B, C9orf127, NGX6	472	ubiquitous	vesicles	n.d.	tumor suppression	n.d.		(72, 73, 74)
TMEM8C/Myomaker	MYMK, TMEM226, TMEM8C	221	myoblast	PM	earlier lethality	myoblast fusion	n.d.		(75, 76)

## Structures of the CREST Family Members

### Overall structures of the CREST family members

**Fig. 2:**
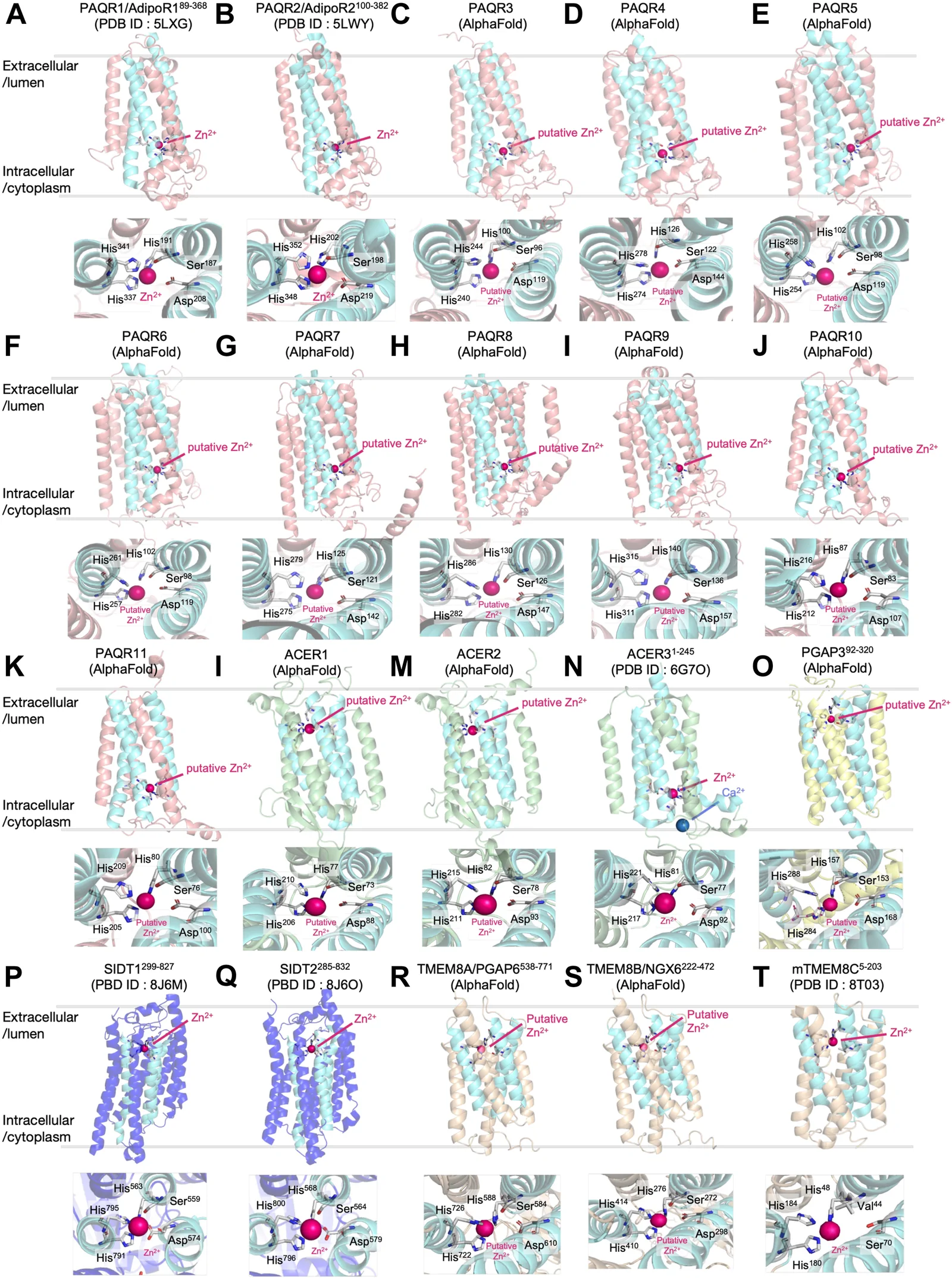
Structures of CREST superfamily members. A-T: With the exception of TMEM8C (mouse) (T), the structures shown are those of human proteins (A-S). The structures of PAQR1/AdipoR1 (A), PAQR2/AdipoR2 (B), and ACER3 (N) were obtained by X-ray crystallography; those of ACER3 (N), SIDT1 (P), SIDT2 (Q), and TMEM8C (T) were by cryo-EM; and the remaining structures were generated using AlphaFold. The topology was determined based on UniProt for proteins without experimental validation. In each structure, the transmembrane segments containing the amino acids that form the active site (His x3, Ser, Asp) are represented by cyan ribbons. Below each structure, the structures of the enzyme active site (or putative active site) that binds Zn^2+^ ions are shown. Note that ACER1 (L), ACER2 (M), SIDT1 (P), SIDT2 (Q), and TMEM8A-C (R-T) differ from other CREST superfamily members in that they possess an enzyme active site on the extracellular side. The grey lines above and below each structure indicate the position of the lipid bilayer. For PAQR2/AdipoR2 (B), ACER3 (N), SIDT1 (P), and TMEM8C (T), lipid (or lipid-like) molecules observed between the transmembrane segments are shown. For ACER3 (n), Ca^2+^ has been observed in the intracellular region.

Figure 2 and Table 2 summarize the structures of human CREST family members, based on X-ray crystallography, cryo-EM, or AlphaFold predictions (77). Most CREST superfamily members possess at least seven transmembrane segments (α-helices), although some members possess eight [PAQR5 (mPRγ), PAQR6 (mPRδ), PAQR7 (mPRα), PAQR8 (mPRβ), and PAQR9 (mPRϵ)] (Fig. 2E–I) or eleven (SIDT1 and SIDT2) (Fig. 2P, Q) transmembrane segments. Of these, seven transmembrane segments appear to be well conserved across the CREST superfamily.

**Table 2:** Structural information on CREST family members

Name	PDB ID	The Number of Transmembrane Segments	Position of the Catalytic Site	Topology	Remarks	Reference
PAQR1/AdipoR1	3WXV,5LXG	7	inner	N_in_-C_out_	Zn2+ in the catalytic active site Oleic acid observed in the cavity	(78, 79, 80)
PAQR2/ApidoR2	3WXW,5LWY,6YX9,6YXD				Zn^2+^ in the catalytic active site	(78, 79, 80, 81)
PAQR3	n.d.				The topology has been experimentally validated.	(82)
PAQR4						
PAQR5/mPRγ		8		N_in_-C_in_		
PAQR6/mPRδ						
PAQR7/mPRα						(83)
PAQR8/mPRβ						(31)
PAQR9/mPRε						
PAQR10/MMD2		7		N_in_-C_out_	The topology has been experimentally validated.	(84)
PAQR11/MMD						
ACER1			outer	N_out_-C_in_		
ACER2					The topology has been experimentally validated.	(51)
ACER3	6G7O,6YXH		inner	N_in_-C_out_	Zn2+ in the catalytic active siteMono-olein observed in the cavityCa^2+^ binding on the cytosolic side	(55, 56, 81)
PGAP3/Per-1	n.d.		outer	N_out_-C_in_		
SIDT1	8K13,8J6M,8WOQ,8WOR,8WOS,8WOT	11			Zn2+ in the catalytic active siteOleic acid, phosphatidyl acid (PA) and ceramide observed in the cavity	(85, 86, 87)
SIDT2	8K10,8J6O				Zn^2+^ in the catalytic active site	(87, 88)
TMEM8A/PGAP6	n.d.	7				
TMEM8B/NGX6						
TMEM8C/Myomaker	8T03				Zn^2+^ in the catalytic active sitePhosphatidylcholine (PC) observed in the transmembrene domain	(89)

Based on structural biological analyses to date, as well as predictions using bioinformatic tools, it is anticipated that the N-termini of PAQR1–11 are located on the cytoplasmic side, while their C-termini are situated on either the extracellular side (PAQR1, 2, 3, 4, 10, 11) (Fig. 2A–D, J, K) or the intracellular side (PAQR5, 6, 7, 8, 9) (Fig. 2E–I). Among the PAQR family members, the X-ray crystal structures of PAQR1/AdipoR1 and PAQR2/AdipoR2 have been determined by several groups (78, 79). As predicted, PAQR1/AdipoR1 and PAQR2/AdipoR2 exhibited a membrane orientation opposite to that of G protein-coupled receptors (GPCRs) (78, 90), with the N-terminus facing the cytoplasm and the C-terminus facing the extracellular space. PAQR5–9 was initially proposed as GPCRs that respond to progesterone at the cell surface to exert immediate non-genomic effects in contrast to the genomic effects mediated by nuclear receptors (83, 91). However, it is now accepted that PAQR5–9 are not GPCRs (31, 92), a conclusion consistent with the membrane topology mentioned above.

The CREST superfamily also contains nine members that do not belong to the PAQR family (Fig. 2, Table 1). Alkaline ceramidases (ACER1–3) are lipid-metabolizing enzymes that have been biochemically characterized as ceramidases, which hydrolyze the amide bond of ceramide to produce sphingosine and fatty acid. Like some of the PAQRs, they traverse the membrane seven times. While ACER3 is thought to have its N-terminus on the cytosolic side and its C-terminus in the lumen (Fig. 2N), ACER1 and ACER2 are expected to have opposite topologies (Fig. 2L, M). PGAP3 is a proposed phospholipase A_2_ (PLA_2_) that is involved in fatty acid remodeling of the glycosylphosphatidylinositol (GPI) moiety of GPI-anchored protein (60). It is also considered to be a seven-transmembrane protein, with its N-terminus oriented toward the lumen and its C-terminus facing the cytosol (Fig. 2O). SIDT1 and SIDT2 differ significantly from other CREST superfamily members in that they traverse the cell membrane 11 times and possess two large extracellular domains (85, 88) (Fig. 2P, Q). Of the 11 transmembrane segments, seven (TM3–6 and 9–11) correspond to the seven transmembrane segments present in PAQR family members and ACERs. TMEM8A/PGAP6, TMEM8B/NGX6, and TMEM8C/Myomaker belong to and form the TMEM8 subfamily. They all have seven transmembrane segments (Fig. 2R–T). TMEM8A/PGAP6 and TMEM8B/NGX6 have large extracellular domains that are absent in TMEM8C/Myomaker.

### Catalytic active site

All CREST superfamily members possess a catalytically active domain or a catalytic-like domain located between transmembrane segments (1). These domains consist of three His residues, one Ser residue, and one Asp residue (H3-S-D) (Fig. 1B). Structural analyses using X-ray crystallography have been performed on PAQR1/AdipoR1 or PAQR2/AdipoR2, which revealed that the three histidine residues (located in transmembrane segments 2 and 7), one aspartic acid residue (located in transmembrane segment 3), one serine residue (located in transmembrane segment 2) and a single water molecule appear to hold a zinc ion (Zn^2+^) in the catalytic domain (78). In the two AdipoRs’ models, a hydrolytic mechanism mediated by individual amino acid residues has been proposed (79). The His residue forms a coordinate bond with the Zn^2+^, thereby stabilizing the active site. The Asp residue forms a hydrogen bond with a water molecule that nucleophilically attacks the amide bond of ceramide, thereby activating the water molecule. The Ser residue stabilizes the oxyanion hole by forming a hydrogen bond with the fatty acid carbonyl or assists the reaction by forming a hydrogen bond with a water molecule. The same structure also forms the catalytic center of ACER3, SIDT1, and SIDT2 (55, 86, 88).

The catalytic (or catalytic-like) domains of all PAQR family members and ACER3 are located in proximity to lipids in the cytoplasmic leaflet of the cell membrane lipid bilayer, suggesting that lipids present in the cytoplasmic leaflet may serve as substrates. Interestingly, in ACER1, ACER2, SIDT1, and SIDT2, the Zn^2+^ ion is located near the extracellular or luminal side of the membrane phospholipid surface. PGAP3, TMEM8A/PGAP6, and TMEM8B/NGX6 also possess three histidine residues and one aspartic acid residue and are predicted to have a structure capable of binding Zn^2+^ ions. Based on the structure predicted by AlphaFold, their catalytic site is expected to be located on the extracellular side, or close to the surface of the luminal membrane lipids. In TMEM8C/Myomaker, Asp and Ser residues are replaced by Ser and Val, respectively. However, according to cryo-EM structures, a Zn^2+^ ion is present at the putative catalytic site on the extracellular side (89). These results strongly suggest that all PAQR family members and ACER3 utilize lipid molecules on the cytoplasmic leaflet, whereas ACER1, ACER2, PGAP3, TMEM8C/Myomaker, SIDT1, and SIDT2 likely utilize lipid molecules on the extracellular or luminal side as substrates.

### Structure of the cavity (pocket) and the presence of lipophilic molecules

In transmembrane proteins, cavities within the membrane play a crucial role in substrate recognition and enzymatic activity, serving as pockets for substrates. As mentioned earlier, recent advances in the structural analysis of CREST superfamily members have revealed characteristic pocket-like structures between the transmembrane domains.

**Fig. 3:**
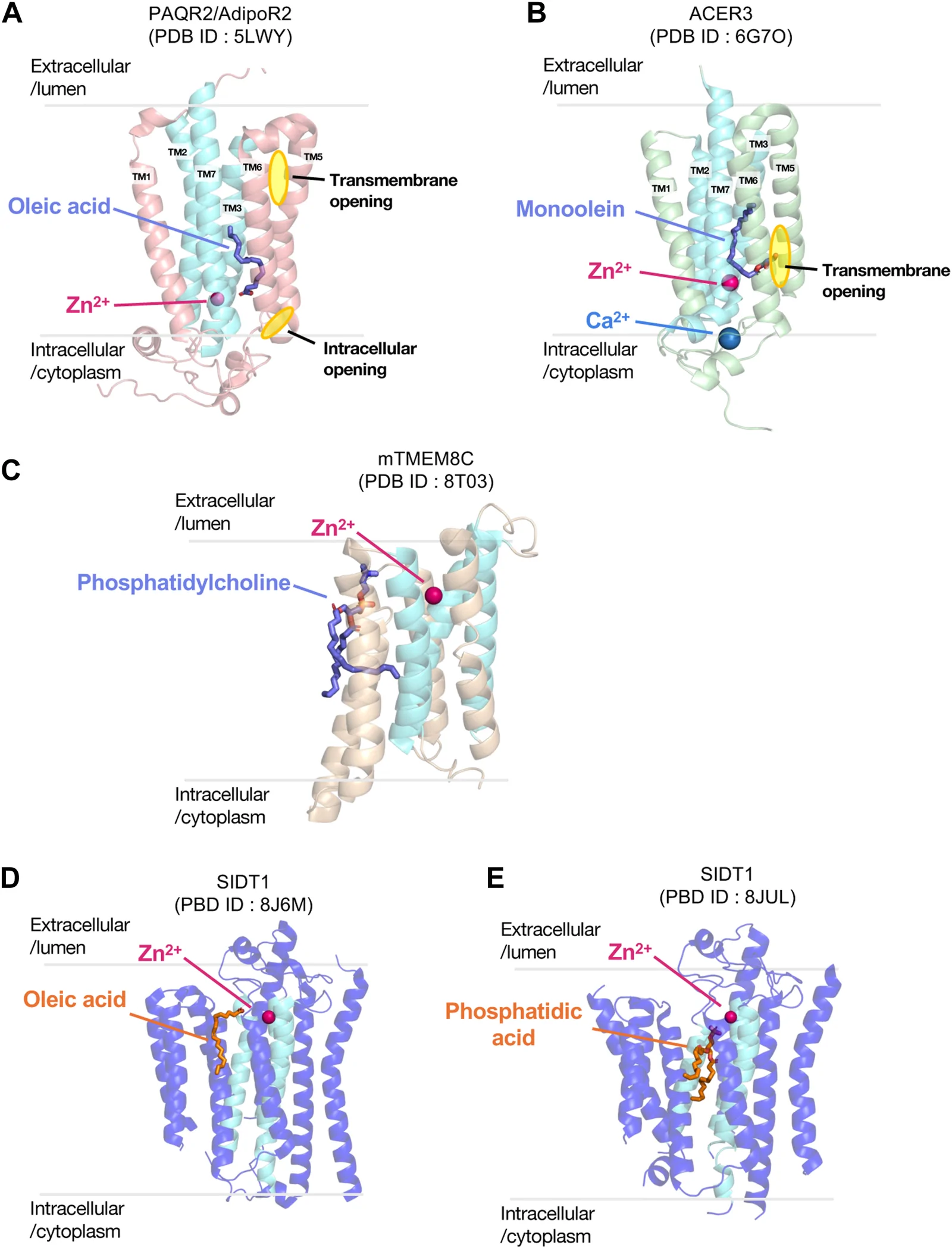
Lipid-binding structure of CREST superfamily members. A–E: Structural models of PAQR2/AdipoR2 (A), ACER3 (B), TMEM8C (C), and SIDT1 (D, E) showing lipid molecules bound within their transmembrane domains. Lipid molecules are displayed in their experimentally determined structural conformations. Yellow ellipses in (A) and (B) indicate openings of the lipid-binding cavity toward either the lipid bilayer or the cytosol. Metal ions (Zn^2+^ or Ca^2+^) that have been observed to interact with each protein are shown as spheres.

In PAQR1/AdipoR1 and PAQR2/AdipoR2, accumulating evidence suggests that free fatty acid-like molecules are held between the transmembrane domains. In initial structural analyses, the electron density observed within the pocket was too weak to allow for the modelling of the free fatty acid (78); however, subsequent model reconstruction revealed that oleic acid binds within the pocket of PAQR2/AdipoR2, and computational methods further suggest that C18:1 ceramide could bind in a similar configuration (79) (Fig. 3A). On the other hand, PAQR1/AdipoR1 was shown to hydrolyze ceramide, but the presence of free fatty acids has not been clearly confirmed (Fig. 2A). These pockets open toward both the extracellular/luminal and cytoplasmic sides of the lipid bilayer, suggesting that substrates and products enter and exit through these openings.

From an X-ray crystal structure, the substrate pocket is also present in ACER3, forming an opening between TM5 and TM6 that provides access to the outer leaflet of the lipid bilayer (55). Furthermore, the binding of a monoolein is predicted within this pocket, which is consistent with the known substrate specificity of ACER3 (56) (Fig. 3B). In addition, the spatial constraints of the pocket suggest that bulky lipids such as large sphingomyelin, glycosphingolipids, and ceramide-1-phosphate are unsuitable as substrates, providing rational structural information for substrate selectivity of the enzyme.

TMEM8C/Myomaker contains a lipid-binding pocket that opens laterally toward the lipid bilayer between TM5 and TM6, where a phospholipid-like electron density has been observed (Fig. 3C) (89). This phospholipid-like molecule is located in the outer leaflet of the lipid bilayer and is positioned adjacent to the zinc-binding site of TMEM8C/Myomaker. Mutation of amino acid residues constituting the lipid-binding pocket markedly impaired myomaker-mediated cell fusion, suggesting that lipid binding is essential for Myomaker function. However, the identity of the bound lipid remains unknown, although it was tentatively modeled as phosphatidylcholine (PC), and it has not been demonstrated that TMEM8C/Myomaker possesses enzymatic activity. It is possible that TMEM8C/Myomaker promotes membrane fusion by binding specific phospholipids and locally remodeling the membrane lipid environment to create a fusion-permissive membrane.

SIDT1 possesses two pocket-like structures that can adopt different conformations depending on cholesterol binding (85). In the cholesterol-bound form, the pocket interior is narrow, preventing the entry of water molecules required for hydrolytic activity; however, in the cholesterol-unbound form, the distance between the Asp in the enzyme activity motif and the Zn^2+^ increases, allowing water molecules to enter. This suggests an intriguing model in which cholesterol binding allosterically regulates SIDT1's hydrolytic activity. Furthermore, binding of oleic acid and di-12:0 phosphatidic acid has been observed in the vicinity of the Zn^2+^, likely representing the products of the ceramidase reaction and phospholipase D (PLD)-mediated phosphatidylcholine (PC) hydrolysis, respectively (93, 87) (Fig. 3D, E). Moreover, in PLD-inactive mutants, binding to PC has been confirmed within the pocket, suggesting that PC may act as a substrate. In another structural model of SIDT1, a lipid-like electron density that could accommodate a short-chain (C8) ceramide was observed within the internal cavity (86).

In SIDT2, the presence of an extensive pocket surrounding the H3-S-D motif has also been confirmed (88) (Fig. 2Q). The study proposed that this pocket opens onto the luminal side, thereby ensuring that water molecules always have access to it. This feature is consistent with its function as a hydrolase. Indeed, ceramidase activity towards C18:0 ceramide has also been experimentally demonstrated (88). However, lipid binding within the pocket has not been clearly confirmed by structural analysis (87).

## Potential roles of CREST family members as lipid-metabolizing enzymes

**Fig. 4:**
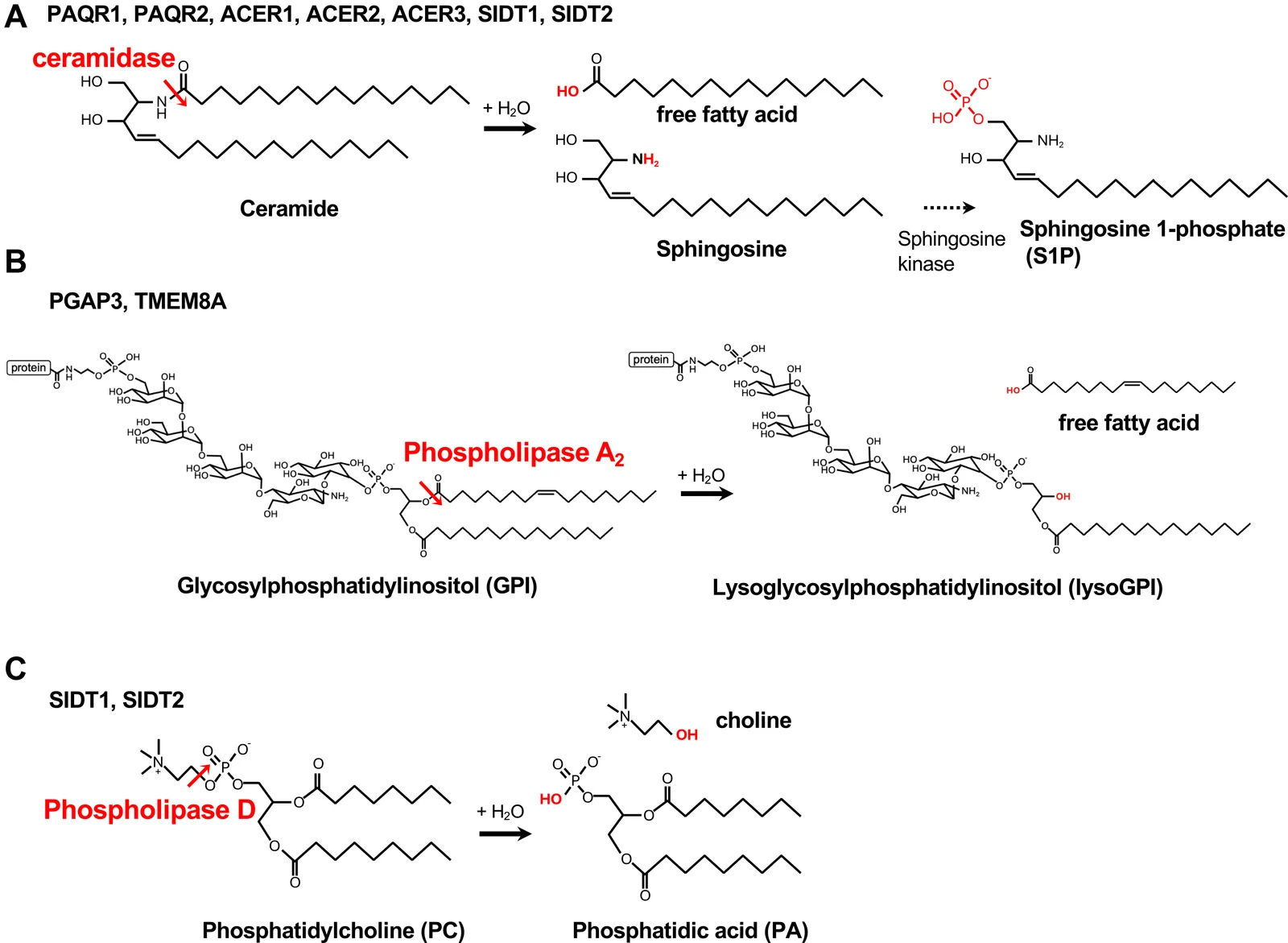
Enzymatic reactions catalyzed by CREST superfamily members. A-C: The figure shows the chemical structures of substrates and products for each reaction, as well as the sites where enzymatic activity is exerted. A: PAQR1/AdipoR1, PAQR2/AdipoR2, ACER1, ACER2, ACER3, SIDT1, and SIDT2 exhibit ceramidase activity. The produced sphingosine is converted into sphingosine-1-phosphate by sphingosine kinase, which then exerts various physiological functions; (B) PGAP3/Per-1 and TMEM8A/PGAP6 possess phospholipase A_2_ activity towards the GPI moiety of GPI-anchored proteins; (C) SIDT1 and SIDT2 possess phospholipase D activity towards phosphatidylcholine.

As mentioned, all CREST superfamily members possess a Zn^2+^-binding mechanism that potentially catalyzes hydrolysis reactions; given the location of their catalytic sites within the cell membrane (Figs. 2 and 3), they may hydrolyze lipids in the cytoplasmic, extracellular, or luminal leaflets. Below, we summarize current knowledge of CREST superfamily members that are potentially involved in lipid metabolism (Fig. 4 and Table 3). With regard to members reported as lipid metabolism enzymes, their biochemical and biological characteristics will also be discussed.

**Table 3:** Enzymatic parameters of CREST family members reported as lipolytic enzymes

name	Substrate	Product	Mutant Validation	Km(μM)	kcat(/s)	Vmax	Remarks	Reference
PAQR1/AdipoR1	C18:0 ceramide	sphingosine, stearic acid	H141R,H191R	n.d.	n.d.	n.d.	Exhibits ceramidase activity toward C6, C18, and C24 ceramides.	(79)
PAQR2/ApidoR2	C18:0 ceramide	sphingosine, stearic acid	H152R,H202R	15.6	0.49 × 10ˆ-3	n.d.	・Exhibits the highest activity toward C18 ceramide (also shows activity toward C6 and C24 ceramides). Its activity is enhanced approximately 20-fold by adiponectin.	(79)
ACER2	C24:1 ceramide	sphingosine	n.d.	83.4	n.d.	29.07 pmol/min/mg	Homogenates from ACER2-overexpressing yeast (Δypc1Δydc1) were used as the enzyme source.・unsaturated long-chain ceramides(e.g. C18:1,C20:1,C20:4) are the best substrates for ACER2.	(51)
ACER3	NBD-C12-PHC(fluorescently labeled ceramide analog)	NBD-fatty acid	H81A,H217A,H221A,D92A,S77A/C	15.48	n.d.	46.94 pmol/min/mg	Homogenates from ACER3-overexpressing yeast (Δypc1Δydc1) were used as the enzyme source.	(94)
SIDT1	8:0 phosphatidyl choline	phosphatidyl acid	E555Q,H795F	323	4.6 × 10ˆ-3	1.2 μM/s/mg	exhibits weak PLD activity toward DMPC and POPC.	(93)
SIDT2	C18:0 ceramide	sphingosine, stearic acid	H796A-H800A	9	0.12 × 10ˆ-3	2.151 μM/s/mg	exhibits weak PLD activity.	(60, 93)

### PAQR1/AdipoR1 and PAQR2/AdipoR2

PAQR1/AdipoR1 and PAQR2/AdipoR2 were discovered as receptors for a ‘beneficial’ adipokine, adiponectin, by Kadowaki and his colleagues in 2003 (90). In 2015, Tanabe *et al.* conducted X-ray structural analyses, demonstrating that they are seven-transmembrane proteins and possess active catalytic-like domains that coordinate a Zn^2+^ ion between the transmembrane segments, thereby leading to the intriguing and compelling hypothesis that these receptor proteins also possess catalytic activity (78).

In 2011, Holland *et al.* suggested that AdipoRs are involved in the induction of ceramidase activity by adiponectin (80). In 2017, Vasiliauskaité-Brooks *et al.* proposed that PAQR2/AdipoR2 possesses ceramidase activity, based on computer simulations, and suggested that PAQR1/AdipoR1 also possesses similar ceramidase activity (79) (Fig. 4A). They also reported that the ceramidase activity of PAQR2/AdipoR2 is increased by approximately 20-fold by its ligand, adiponectin. However, the *K*cat value for basal ceramidase activity was extremely low at 0.49 × 10^−3^ s^−1^, indicating that one PAQR2/AdipoR2 molecule would hydrolyze one ceramide molecule in approximately 30 min (Table 3). Even if activity is increased by the addition of adiponectin, PAQR2/AdipoR2 would still only possess the ability to hydrolyze one ceramide molecule every few minutes, which is an extremely low level of activity for a typical enzyme. In light of these results, Vasiliauskaité-Brooks *et al.* also mentioned the presence of other substrates (79). Thus, it is possible that AdipoRs have lipid hydrolytic activities other than ceramidase activity.

Meanwhile, Marc Pilon’s group proposed that PAQR2/AdipoR2 in *C. elegans* has a role in preventing a reduction in cell membrane fluidity caused by palmitic acid loading (95). It is well known that saturated fatty acids, such as palmitic acid, induce endoplasmic reticulum stress (96, 97) and reduce cell membrane fluidity (95), thereby causing saturated fatty acid toxicity. They reported that in *C. elegans*, PAQR2/AdipoR2 mutants exhibited reduced cell membrane fluidity upon palmitic acid loading, accompanied by an increase in palmitic acid-containing phospholipids (95). Subsequently, they demonstrated that similar phenomena occur in mammalian cells and proposed that PAQR2/AdipoR2 specifically contributes to the homeostasis of cell membrane phospholipids when saturated fatty acids, such as palmitic acid are oversupplied (98, 99). Although the group has made little mention of the function of PAQR1/AdipoR1, they have shown that double mutants of PAQR1/AdipoR1 and PAQR2/AdipoR2 exhibit a further reduction in cell membrane fluidity, suggesting that PAQR1/AdipoR1 possesses a function similar to that of PAQR2/AdipoR2 (99). They also proposed a model for the mechanism by which PAQR2/AdipoR2 maintains cell membrane fluidity: sphingosine produced by PAQR2/AdipoR2's ceramidase activity is converted into sphingosine 1-phosphate (S1P), which, via S1P receptors, activates fatty acid desaturases, thereby increasing membrane phospholipid unsaturation (99). Although this hypothesis is really intriguing, another mechanism is possible that PAQR1/AdipoR1 and PAQR2/AdipoR2 act on other substrates, such as saturated fatty acid-containing phospholipids including palmitic acid, to suppress cell membrane saturation, since the ceramidase activities of PAQR1/AdipoR1 and PAQR2/AdipoR2 are quite low, as mentioned above.

There is also ample evidence that PAQR1/AdipoR1 is involved in lipid metabolism. Several reports have demonstrated that PAQR1/AdipoR1 KO mice exhibited a retinitis pigmentosa phenotype (3, 5, 100, 101, 102). In addition, Rice *et al.* (5) and Osada *et al.* (102) showed that phospholipids containing very long-chain polyunsaturated fatty acids (VLC-PUFAs) and docosahexaenoic acid (DHA) were specifically reduced in the PAQR1/AdipoR1 KO retina. Based on these findings, Rice *et al.* proposed that PAQR1/AdipoR1 has a role as a transporter for DHA.

Several in vitro studies have indicated that both PAQR1/AdipoR1 and PAQR2/AdipoR2 form both dimer and heterodimer (4, 103). The oligomer formation may affect internalization, localization, and downstream signaling. However, precise roles of the oligomer formation remain to be determined.

### PAQR4

PAQR4, unlike PAQR1/AdipoR1 and PAQR2/AdipoR2 described above, does not respond to adiponectin (2). Recently, several studies have suggested a role for PAQR4 in ceramide metabolism. Pedersen *et al.* reported that ceramidase activity was reduced in lysates of PAQR4-knockdown cells compared with controls, and that overexpression of PAQR4 increased levels of sphingosine-1-phosphate (S1P), indicating that PAQR4 possesses ceramidase activity (17). PAQR4 is highly expressed in lung cancer cells and is thought to contribute to the evasion of ceramide-induced apoptosis through its ceramidase activity. Further verification is required regarding the ceramidase activity of PAQR4, since Zhu *et al.* reported that PAQR4 itself does not exhibit ceramidase activity; instead, PAQR4 interacts with ceramide synthases (CERS) 2 and 5 to enhance their stability, thereby promoting ceramide production and contributing to the regulation of ceramide levels in adipocytes (18).

### PAQR8/mPRβ and PAQR9/mPRε

PAQR8/mPRβ and PAQR9/mPRε, together with the other three mPRs [mPRγ (PAQR5), mPRδ (PAQR6), mPRα (PAQR7)], have been reported to respond to progesterone. They are expected to be the receptors that mediate progesterone functions independent of nuclear progesterone receptors (83, 104, 105).

PAQR8/mPRβ has been suggested not to function as a classical GPCR, as progesterone stimulation does not alter intracellular Ca^2+^ or cAMP levels. On the other hand, progesterone induces ERK phosphorylation via PAQR8/mPRβ and promotes neurite outgrowth in neuronal cells (31). In addition, in oocytes, mPRβ has been reported to induce clathrin-dependent endocytosis upon progesterone stimulation, interact with APPL1 and Akt2, and thereby promote meiotic resumption (32). Interestingly, PAQR8/mPRβ has also been shown to function as a co-receptor with the lipid hydrolase ABHD2 (α/β hydrolase domain-containing protein 2) to induce oocyte maturation through the regulation of lipid metabolism (106). Indeed, progesterone treatment of oocytes results in PAQR8/mPRβ- and ABHD2-dependent decreases in glycerophospholipids and sphingolipids, accompanied by increases in lipid mediators such as lysophosphatidic acid (LPA) and prostaglandins. These findings suggest that such lipid mediators may act through GPCRs to propagate downstream signaling. Furthermore, mutation of the putative catalytic active site of PAQR8/mPRβ to alanine does not impair progesterone-induced meiotic resumption (106), indicating that the intrinsic enzymatic activity of PAQR8/mPRβ is dispensable for this process, at least in this context.

The physiological functions of PAQR9/mPRε in the liver include the regulation of fasting-induced ketogenesis and fatty acid oxidation (36). Meanwhile, PAQR9/mPRε in the pancreas contributes to blood glucose regulation and lipid homeostasis in diabetic mice (37). However, the relationship between these phenotypes and progesterone, a ligand for PAQR9/mPRε, has not yet been fully elucidated. Watanabe *et al.* recently conducted a comprehensive lipidomics study of PAQR9/mPRε in white adipose tissue (WAT) and reported a decrease in arachidonic acid metabolites, including prostaglandins, and a reduction in phospholipids containing arachidonic acid in knockout mice, suggesting that PAQR9/mPRε influences phospholipid and fatty acid metabolism (107).

### PAQR11/MMD

PAQR11/MMD has been identified as a gene whose expression increases significantly during monocyte-to-macrophage differentiation. It has recently been reported that PAQR11/MMD regulates lipolysis in adipose tissue during fasting (42). Under fasting conditions, the expression of PAQR11/MMD is reduced. This leads to suppression of phosphodiesterase 4D (PDE4D) degradation, which negatively regulates cAMP levels, via the SKP1–CUL1–FBXO2 E3 ubiquitin ligase complex. As a result, cAMP signaling is enhanced, leading to the activation of hormone-sensitive lipase (HSL), a key executor of triacylglycerol hydrolysis, as well as the phosphorylation of perilipin1 (PLIN1), which promotes the activation of adipose triglyceride lipase.

Furthermore, PAQR11/MMD functions as a scaffold protein for ACSL4 and LPLAT11/MBOAT7 (43). ACSL4 is an enzyme that converts PUFAs, such as arachidonic acid, into acyl-CoAs (108). LPLAT11/MBOAT7 is an enzyme that uses arachidonyl-CoA, produced by ACSL4, as a substrate to introduce arachidonic acid into another substrate, lysophosphatidylinositol (LPI) (109, 110, 111). Consequently, PAQR11/MMD, together with ACSL4 and LPLAT11/MBOAT7, appears to contribute to the production of arachidonic acid-containing phosphatidylinositol (PI), which is a major PI molecular species in the body. However, the role of PAQR11/MMD's catalytic activity remains unclear at present.

### Alkaline ceramidases

In ceramide metabolism, ceramidases are key enzymes that convert ceramide into sphingosine. In humans, five distinct ceramidase genes have been cloned, each encoding a different ceramidase. These are acid ceramidase (AC), neutral ceramidase (NC), alkaline ceramidase 1 (ACER1), alkaline ceramidase 2 (ACER2), and alkaline ceramidase 3 (ACER3), and are classified according to the optimal pH of each enzyme. The three isoforms of mammalian alkaline ceramidase—ACER1–3—belong to the CREST superfamily (46). It has been reported that ACER1 is expressed in the endoplasmic reticulum, ACER2 in the Golgi apparatus, and ACER3 in both the endoplasmic reticulum and the Golgi apparatus (46). Based on their localization, it is thought that acidic and neutral ceramidases hydrolyze ceramide in the extracellular space or on the luminal leaflet, whereas alkaline ceramidase hydrolyzes ceramide on the luminal leaflet (ACER1, ACER2) or the cytoplasmic leaflet (ACER3). Ceramidases hydrolyze ceramide to produce sphingosine, which is then converted into sphingosine 1-phosphate (S1P) (Fig. 4A). The pathophysiological roles of each ceramidase have been elucidated through functional analyses using knockout mice and other models (112).

### PGAP3

One mode of membrane binding for membrane proteins is the GPI anchor. In the GPI structure, the protein is bound to the inositol ring of PI via glucosamine, mannose, and ethanolamine (113). Not only do the GPI molecules in the GPI anchor and normal PI differ in their location, but their fatty acid composition also differs significantly (113). GPI-anchored proteins are located on the extracellular or lumen-facing leaflet, and their GPI molecules are rich in saturated fatty acids; this is thought to be important for the biased localization of GPI-anchored proteins to the lipid rafts, which are rich in sphingolipids and cholesterol. In contrast, PI in cells is rich in unsaturated fatty acids, such as arachidonic acid, and is mainly located in the cytoplasmic leaflet. Based on these findings, it was hypothesized that PI synthesized in the ER is subjected to fatty acid remodeling during the process of its conversion to GPI. Kinoshita’s group identified PGAP3 as the enzyme involved in this fatty acid remodeling of GPI. The group proposed that unsaturated fatty acids at the *sn*-2 position of PI in GPI anchor precursors—which are biosynthesized in the endoplasmic reticulum and attached to proteins—are removed by PGAP3, resulting in a lysophospholipid intermediate, which is subsequently replaced by saturated fatty acids (61, 113). Although direct enzymatic activity has not been demonstrated, PGAP3 is thought to be a GPI-specific PLA_2_ (Fig. 4B).

### TMEM8A/PGAP6 and TMEM8B/NGX6

The aforementioned group, led by Kinoshita, demonstrated that TMEM8A/PGAP6 cleaves the fatty acid of the GPI-anchor region of CRIPTO, a GPI-anchored protein, thereby promoting the extracellular release of CRIPTO, and that this release is essential for embryonic development. Consequently, TMEM8A/PGAP6 is considered to be a GPI-specific PLA_2_ that promotes the extracellular release of GPI-anchored proteins (70, 71) (Fig. 4B).

TMEM8B/NGX6 was first reported to influence the expression of cell adhesion-related proteins in nasopharyngeal carcinoma (NPC) cells and to have a role as a tumor-suppressing molecule (72). It also suppresses the Wnt–β-catenin signaling pathway in several cancer cell lines, leading to decreased cell viability and reduced invasive capacity (73, 74). However, many details of its biochemical functions remained unclear. Lee *et al.* created a chimeric protein by fusing the N-terminal region of TMEM8A/PGAP6 with the transmembrane domain of TMEM8B/NGX6 and demonstrated that it exhibits PLA_2_ activity towards GPI-anchored proteins comparable to that of TMEM8A/PGAP6 (70). The finding suggests that the transmembrane domain of TMEM8B/NGX6 contributes to its hydrolytic activity. However, no direct experimental evidence has yet been obtained regarding the hydrolytic activity of TMEM8B/NGX6 alone. Therefore, further detailed biochemical and structural biological analyses are required to determine whether TMEM8B/NGX6 possesses enzymatic activity.

### TMEM8C/myomaker

TMEM8C/Myomaker is a molecule essential for muscle cell fusion and possesses the ability to promote the fusion of two contacting cell membranes (114). As mentioned above, although TMEM8C/Myomaker lacks an Asp and a Ser in its catalytic-like domain, it binds a Zn^2+^ ion within its structure, similar to other CREST superfamily molecules. Cryo-EM analysis has shown that TMEM8C/Myomaker forms a homodimer, and mutational analysis of the amino acid residues at the dimer interface has revealed that dimerization is essential for TMEM8C/Myomaker's cell-fusion ability (89). TMEM8C/Myomaker is a protein expressed on the cell membrane surface, and the putative catalytic site may hydrolyze lipid molecules in the extracellular leaflet (Fig. 2T). Cryo-EM analysis suggests that a single phospholipid molecule is bound on the extracellular side of TMEM8C/Myomaker (89). Since introducing mutations into this putative phospholipid-binding site significantly reduced the cell fusion ability of TMEM8C/Myomaker, it is concluded that phospholipid binding by TMEM8C/Myomaker is necessary for cell fusion (89). Taken together, these findings suggest that TMEM8C/Myomaker interacts with lipids in the extracellular leaflet and, furthermore, is likely to exert its function by hydrolyzing these lipids. However, whether TMEM8C/Myomaker hydrolyzes lipids remains unclear.

Very recently, Wherley *et al.* reported that ether-containing phospholipids such as ether PE are elevated in TMEM8C/Myomaker-expressing cells, which promotes the membrane fusion event, indicating a role for the protein in remodeling plasma membrane lipid distribution (115). Although the role of the active site is unclear in this study, the authors discuss its influence on fatty acid dynamics.

### SIDT1 and SIDT2

SID-1 was identified in *C. elegans* as a factor involved in the uptake of dsRNA to induce RNA interference (RNAi) (116). However, it has been controversial whether the human SID-1 homologs, SIDT1 and SIDT2, are involved in dsRNA uptake (85, 117). It has been experimentally demonstrated that SIDT1 and SIDT2 lack dsRNA uptake activity (87). Consistent with this, structural analyses have suggested that the transmembrane region of SIDT1 is too narrow to accommodate dsRNA for transport (85). Furthermore, SIDT1 and SIDT2 share a cholesterol recognition amino acid consensus (CRAC) domain with ChUP-1, which mediates dietary cholesterol uptake in *C. elegans*, and have also been reported to function as cholesterol transporters (62). On the other hand, as mentioned above, SID-1, SIDT1, and SIDT2 can hydrolyze lipid molecules in the extracellular leaflet. To date, several groups have reported that SIDT1 and SIDT2 possess ceramidase activity (85, 88) (Fig. 4A). Furthermore, there are reports that SIDT1 binds to phosphatidic acid (PA) and exhibits PLD activity toward PC (93) (Fig. 4C). The *K*cat of its PLD activity toward di-8:0 PC is 4.6 × 10^−3^ s^−1^, which is markedly lower than that of other PLDs (e.g., ∼5508 s^−1^ for plant PLD, ∼110 s^−1^ for *Streptomyces klenkii* PLD, and ∼68 s^−1^ for *Streptomyces hiroshimensis* PLD) (118, 119, 120) (Table 3). Furthermore, its activity toward native membrane phospholipids such as di-myristoyl PC (DMPC) and palmitoyl-oleoyl PC (POPC) is even lower, being approximately one-fifth and one-tenth of that toward di-8:0 PC, respectively. These observations raise the question of whether the PLD activity of SIDT1 is functionally relevant under physiological conditions and warrant further investigation. Similarly, PLD activity against di-8:0 PC has been reported for SIDT2 (93). The physiological significance of ceramidase activity in SIDT1 and SIDT2 remains largely unclear at present. Future detailed analyses will shed light on the role of these molecules as ceramidase. Both SidT1 and SidT2 form homodimers. The structure of the dimer interface changes depending on the presence or absence of cholesterol binding, so this dimerization may affect the binding of lipid molecules (85).

## Conclusion

Most CREST superfamily members are thought to possess lipid-hydrolytic activity. However, there are few reports demonstrating lipid hydrolytic activity biochemically, and even when detected, it has been extremely low. This may be due to the fact that standard in vitro enzyme activity assays typically rely on detergent micelles, in which membrane proteins and lipid substrates are mixed under conditions that do not fully recapitulate the native membrane environment. Moreover, functional redundancy may also mask alterations in lipid metabolism in mutant conditions. Indeed, knockdown of ACER3 results in upregulation of ACER2 mRNA expression, suggesting that ACER2 may compensate for the loss of ACER3 function (56). Further biochemical characterization of CREST superfamily members, particularly using lipidomic approaches, will be important for elucidating their physiological functions.

## Conflict of interest

The authors declare that they have no conflicts of interest with the contents of this article.
